# Strontium-Doped Hematite as a Possible Humidity Sensing Material for Soil Water Content Determination

**DOI:** 10.3390/s130912070

**Published:** 2013-09-10

**Authors:** Jean-Marc Tulliani, Chiara Baroni, Laura Zavattaro, Carlo Grignani

**Affiliations:** 1 Department of Applied Science and Technology, Politecnico di Torino, C.so Duca degli Abruzzi 24, 10129 Torino, Italy; E-Mail: chiara.baroni@polito.it; 2 Department of Agricultural, Forest and Food Sciences, Università di Torino, Via Leonardo da Vinci, 44, 10095 Grugliasco, Italy; E-Mails: laura.zavattaro@unito.it (L.Z.); carlo.grignani@unito.it (C.G.)

**Keywords:** hematite, strontium hexaferrite, humidity sensor, soil water content determination

## Abstract

The aim of this work is to study the sensing behavior of Sr-doped hematite for soil water content measurement. The material was prepared by solid state reaction from commercial hematite and strontium carbonate heat treated at 900 °C. X-Ray diffraction, scanning electron microscopy and mercury intrusion porosimetry were used for microstructural characterization of the synthesized powder. Sensors were then prepared by uniaxially pressing and by screen-printing, on an alumina substrate, the prepared powder and subsequent firing in the 800–1,000 °C range. These sensors were first tested in a laboratory apparatus under humid air and then in an homogenized soil and finally in field. The results evidenced that the screen printed film was able to give a response for a soil matric potential from about 570 kPa, that is to say well below the wilting point in the used soil.

## 1. Introduction

It is well known that only 2.5% of global water resources is potentially available for human, animal and plant consumption, while the remaining 97.5% resides in the oceans [1]. Water for irrigation constitutes one of the greatest pressures on freshwater resources. Agriculture accounts for about 70% of global freshwater withdrawals (reaching up to 90% in some fast-growing economies), while it is generally reported that approximately 20% of the world's freshwater withdrawals are used by industry, although this varies between regions and countries, and 10% by the municipal sector [2]. Global population growth projections of 2 to 3 billion people over the next 40 years, combined with changing diets, result in a predicted increase in food demand of 70% by 2050 [2]. Thus, water demand will increase too. Moreover, water is needed also to produce and make use of energy: all forms of energy require water at some stage of their life cycle, which includes production, conversion, distribution and use. Global energy consumption is expected to increase by about 50% between 2007 and 2035, to meet rising populations and living standards [2]. Climate change further complicates the issue: the fast alternate cycles of floods (in Central Europe, South of China and other Asian countries in 2002 and 2011) and long periods of drought (in Europe in 2003 and in the Horn of Africa in 2011) are susceptible to lead to emerging crisis and regional conflicts because of food scarcity.

In such a context, a substantial increase of irrigation water productivity, of investments in the existing irrigation systems modernization, and of new water resource developments are clearly needed. The automation of irrigation in modern farms, greenhouses or buildings has expanded over the last years, in order to increase the irrigation efficiency and to reduce the maintenance expenses. Frequently, automatic irrigation systems are based on prescheduling, which does not account for the exact conditions. Thus, water wasting may follow a sudden rain or crop losses can result from unexpected dry atmosphere conditions. For a good irrigation practice, water should be applied to the soil at the right time and amount, to avoid both crop stress (and consequent yield reduction) and losses due to deep percolation and runoff. As a consequence, measurements of the water content in soil should be used for evaluating the level of water reserve.

The existing irrigation systems are based on four categories of basic sensors, used for irrigation scheduling [3,4]:

(a) Tensiometers, which measure soil water potential through a ceramic cup which is inserted into the soil and establishes an equilibrium with the soil water tension up to *ca.* 0.8 bar;
(b) Gypsum blocks, which measure soil water content and work on the simple principle of electrical resistance measurement, better suited to dry soils than wet soils;
(c) Neutron probes, which measure soil water content through detecting interaction of water and neutrons emitted by a radioactive source; these need to be calibrated for each type of soil and are subject to specific norms that limit their use in most countries;
(d) Methods that directly or indirectly measure the soil dielectric constant: time domain reflectometry (TDR), that measures the propagating velocity of an electro-magnetic pulse travelling along the sensor; and capacitance, that measures the resonance frequency of a circuit, in which the probe itself is used as a capacitor [3]. Thus, when the amount of water changes in the soil, the probe will measure a change in capacitance that can be directly correlated with a change in water content [3]. Several commercial sensors have been developed on the basis of these relationships.

All these methods are discontinuous, quite expensive or subjected to accelerated deterioration, often require a soil-specific calibration, and are sometimes complex, difficult to install, and results must be evaluated by experts. Finally, the variability of soil environment and the influence of water content on different chemical and physical properties of soils make all measurements carried out so far problematic to analyze in a proper way, since they are affected by uncertainty and they are related to few soil typologies. Thus, measurement of soil water content is still an unsolved technical problem. The necessity to develop new humidity sensors that do not possess the above mentioned drawbacks is then obvious.

Soil is comprised of minerals, soil organic matter, water and air. The composition and proportion of these components greatly influence soil physical properties, including texture, structure, and porosity, the fraction of pore space in a soil. In turn, these properties affect air and water movement in a soil, and thus the soil's ability to function and sustain productivity [3]. Water in soil occupies pore spaces that arise from the physical arrangement of the particulate solid phase, competitively and often concurrently with the soil gas phase [3]. The porosity in the soil generally lies in the range 0.3 L/L (in coarse sandy soils) to 0.6 L/L (in fine clayey soils) [3]. Water evaporation depends on soil properties and on atmospheric conditions; in a saturated soil, the free water in larger pores evaporates first, the remaining water is retained mainly in small soil pores by strong capillary forces, and vapor pressure of the air in equilibrium with the pore water will therefore be lower than in the air close to a free water surface [5]. Therefore, the devices used for measuring the atmospheric moisture have been also proposed for soil water content determination. These sensors are based on electrical resistance measurements of porous materials such as polymers and ceramics [6,7]. Ultrasonic waves have been also envisaged for this aim [8].

A sensor to measure soil air relative humidity should exhibit several characteristic: it should be small and rugged so that it can be inserted in soil without significantly affecting the surrounding porous medium, while also being able to resist damage by adjacent soil particles. The sensor should be relatively insensitive to salinity, which can be relevant in semiarid and arid regions. Hysteresis should be minimal. It should be able to withstand or compensate for change in temperature, and should be inexpensive so that multiple sensors can be installed. The sensors should also be able to measure relative humidity values over a broad range, ideally 0–100% and not require calibration for different soil types [7].

It is well known in the literature that ceramic humidity sensors features are linked to bulk and surface properties, specifically, pore size distribution, mean grain size and doping. The materials composition, their microstructure and porosity can strongly influence their electrical conductivity and the presence of active catalytic sites, thus their functional properties [9–12]. Hematite and doped-hematite are known gas and humidity sensors [13–16]. The humidity properties of pure and doped α-Fe_2_O_3_ and of ferrites have been studied in detail throughout the years: iron oxide [17,18], MgFe_2_O_4_ [19,20], Sn^4+^ or Mo^6+^-doped MgFe_2_O_4_ [21], MgFe_2_O_4_-CeO_2_ [22], Li-doped Fe_2_O_3_ [23,24], Zn^2+^ and Au^3+^-doped Fe_2_O_3_ [25], K, Na, Mg, Ca, Rb, Ba, Sr-doped Fe_2_O_3_ [24], Au-doped LiFe_2_O_3_ [26], Mg-Cu ferrite [27], Li substituted Mg-ferrite [28], LaFeO_3_ [29], La_0.8_Sr_0.2_Fe_1-x_Cu_x_O_3_ [30], Fe_2_O_3_ nanoparticles on sepiolite [31,32], K^+^-β-ferrite [33], Mn-Zn ferrite [34] and silica-Fe_2_O_3_ [35] were already evaluated as humidity sensors.

Much interest has been focused on study of Mg-based ferrite as humidity sensitive material since it is inexpensive and robust [27]. However, in the Sigma-Aldrich catalogue, strontium carbonate is about seven times more expensive than magnesium carbonate, nevertheless, due to the small amount used in this study, we believe that strontium-doped hematite humidity sensors are still competitive, from an economical point of view, with respect to other ferrites. Therefore, the scope of this work is to study the sensing behavior of Sr-doped hematite for soil water content determination. To this aim, the electrical response of this material was first studied in air, then in a simulated soil and finally, in field.

## 2. Experimental Section

**Figure 1.**
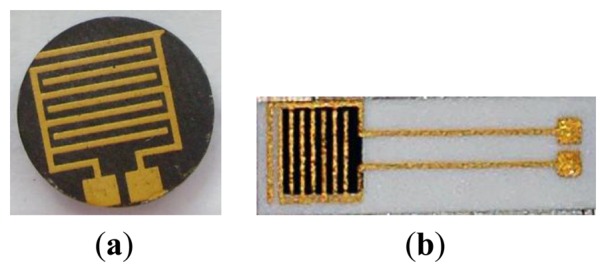
(**a**) Different investigated sensor geometries: pellet; (**b**) Screen printed on planar substrate.

α-Fe_2_O_3_ powder (Aldrich > 99%, particle size distribution below 2 μm) was mixed in ethanol with strontium carbonate used as a precursor of 5 wt% equivalent of strontium oxide respect to hematite (Fluka > 99%), in a planetary mill for 1 h. After drying overnight, the mixture was uniaxially pressed at 370 MPa and calcined at 900 °C for 1 h. The pellets were then manually ground by means of an agate mortar and an agate pestle, prior to be planetary milled for 6 h in ethanol, in order to reach the same particle size distribution as the starting hematite. After drying overnight, the powder was ready to use. Then, pellets were pressed again in the same conditions above mentioned and fired in the temperature range 800–1,000 °C. Finally, gold electrodes (ESL 520 A) were screen printed on the surface of the pellets and after drying overnight, all the sensors were fired at 520 °C for 18 min, according to the ink's manufacturer recommendations (Figure 1a).

The screen-printed sensors were prepared according to [24]: the ink, based on Sr-doped α-Fe_2_O_3_ powder, was deposited on an α-alumina substrate (Coors Tek, ADS-96 R, 0.85 cm × 5.1 cm) through a 270 mesh steel screen (Figure 1b). The thick films, whatever the firing temperature, presented a poor adhesion to the substrates, then gold electrodes deposition could be done without damage only on unfired films and sensors were heat treated at 900 °C for 1 h, with a 5 °C/min heating and cooling ramp. The interdigitated electrodes illustrated in Figures 1a and b are included in an area of about 0.6 cm^2^ and the fingers have a width of 380 ± 25 μm and are distant each other of 460 ± 18 μm (average values determined from ten measurements on SEM images). Finally, each comb is distant of 210 ± 28 μm from the other. The sensing films (Figure 1b) occupy an area of 0.4 cm^2^ and have a thickness of about 20–30 μm.

The pellets were characterized by X-Ray diffraction (XRD, Philips PW1710) in the 5–70° 2θ range and by mercury intrusion porosimetry (Carlo Erba Porosimeter 2000) after firing. The screen printed films were observed by means of a Scanning Electron Microscope (SEM, Hitachi S2300) and of a Field Emission-SEM (FE-SEM, Hitachi S4000). Image analysis (Image-Pro Plus 7.0 by MediaCybernetics Inc.) was also performed on 6,000 × magnification images from the SEM microscope to determine pore size distribution and pore area. Pore detection was done image by image, as a function of the contrast of each picture, by adjusting the grey value threshold. Once the pores were highlighted, the system measured the corresponding area of all the thresholded objects and then converted it into an equivalent diameter, assuming that all of them were equivalent to perfect circles. All these diameters were finally “high-passed filtered” by means of a minimum value (0.09 μm) chosen to remove the smallest objects corresponding to pores or parts of pores that were not correctly detected.

The humidity response of the sensors in the 0–100% RH range was studied in a laboratory apparatus at room temperature, under an air flow rate of 0.05 L/s [24]. The relative humidity was increased by steps, each one of three minutes. A commercial humidity and temperature probe was used as a reference for temperature and RH values (Delta Ohm HD2101.1), accuracy: ±0.1% in the 0–100% RH range and −50–250 °C temperature range. Each tested sensor was alimented by an external alternating voltage (V = 3.6 V at the rate of 1 kHz; cost of all the electronic components of this circuit: about 40 euros) and then constituted a variable resistance of this electrical circuit. A multimeter (Keithley 2000) was used to measure the tension V_DC_ at the output of the circuit. The sensor resistance was determined by substituting them, in the circuit, by known resistances and then by plotting a calibration curve R = f(V_DC_).

The sensor response SR was calculated from the sensors resistance value [Equation (1)] [36]:

(1) $$SR=\frac{R(P_{H_{2}O})-R(P_{H_{2}O\to 0})}{R(P_{H_{2}O\to 0})}$$

where *R*(*P_H_*_2_*_O_*) is the total resistance of the material when exposed under *P_H_*_2_*_O_* partial pressure and *R*(*P_H_*_2_*_O_*) is the resistance measured under dry air.

**Figure 2.**
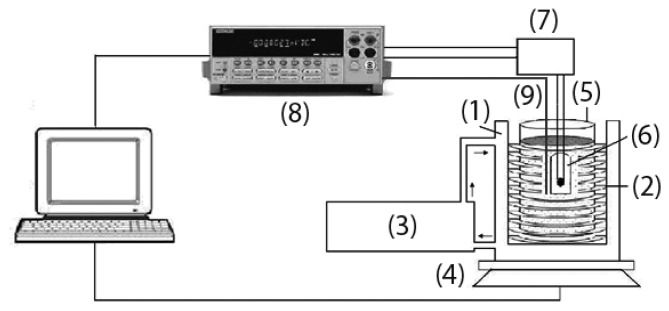
Scheme of the laboratory system for soil water content measurement.

An experimental laboratory system (Figure 2) was also set-up for continuous monitoring of soil mass, by means of an electronic balance connected to a PC, after the addition of a known quantity of water to a 2 mm sieved and homogenized soil and its subsequent progressive evaporation with time. The water content in soil is then given by Equation (2):

(2) $$\text{Water Content}=\frac{{\text{Volume}}_{\text{water}}}{{\text{Volume}}_{\text{soil}}}$$

The soil water content evaluation system was made up of a polystyrene box (1) containing a tube (2) alimented with warm water heated by a thermostated bath (3). The polystyrene box, placed on a balance (Exacta Optech) (4), also hosted a beaker with the soil (5) and the porous cup with the sensor to test (6). The sensor was alimented with the same electrical circuit (7) of the laboratory apparatus for RH testing. A multimeter (Keithley 2700/E) (8) recorded the sensor voltage, which was converted in resistance value through the calibration curve R = f(V_DC_), already used for measurements under air. The decrease in water content was then correlated to the variation of the sensors tension. Finally, the effective temperature of the soil was monitored by means of a Pt100 thermocouple connected to the multimeter (9).

**Figure 3.**
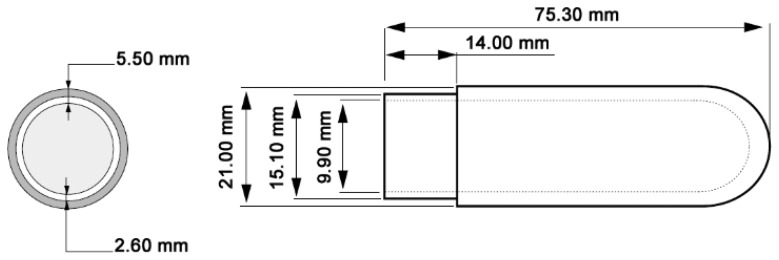
Scheme of the porous SDEC 2150 ceramic cup [38].

During these measurements, humidity sensors inserted in the soil were protected by a porous cup. Thus, two different cups were evaluated, a polyethylene-based one and a ceramic-based one. The former is used for thermohygrometers calibration with saturated salts solutions [37], while the latter is used in tensiometry for matric potential measurements (SDEC 2150, Table 1, Figure 3) [38].

As these measurements lasted several weeks, the balance presented a drift with time which tended to underestimate the exact weight of all the system. Therefore, an equivalent mass of the monitoring system was put regularly onto it and the evolution of the measured constant mass was monitored with time. The drift of the balance was then fitted with a good approximation (R^2^ = 0.998) by a polynomial function of order 6 and was corrected for all experimental measurements performed with the system.

This system allowed us to describe soil humidity in two ways [3,4]:

(a) Gravimetric soil water content, *i.e.*, how much water is in the soil on a weight basis, e.g., 0.3 g water per 1 g of dry soil;
(b) Volumetric soil water, expressed as volume of water by volume of soil, which can be measured if the volume of water and soil are known, or can be calculated by multiplying the gravimetric soil water content by the soil bulk density; it uses units of cubic centimetres (or millilitres) of water per cubic centimetre of soil.

The volumetric soil water content is in relation with the soil water potential through a curve which is called soil water retention curve. The soil water potential is a measure of the tension needed to extract water from soil when it becomes drier and it is expressed in kilopascals (kPa). Potential is also referred to as soil water suction. The soil water retention curve is described by a nonlinear function whose shape greatly depends on the structure and composition of the material constituting the soil [3]. Under equilibrium conditions the soil water potential is equal to the potential of water vapor in the surrounding soil air. The relative humidity is related to the water potential of the vapor through the Kelvin Equation (3) [3]:

(3) $$RH=\frac{e}{e_{0}}=\exp\left[(M_{w}\Psi_{w})/\rho_{w}RT\right]$$

where: *e* is water vapor pressure, *e_0_* is saturated water vapor pressure at the same temperature, *M_w_* is the molecular weight of water (0.018 kg·mol^−1^), *R* is the ideal gas constant (8.314 J·mol^−1^·K^−1^), *T* is absolute temperature, *ρ_w_* is the density of water (1,000 kg/m^3^ at 20 °C). Often, the entire range of plant-available water is such that 98 < RH% < 100. In these conditions, Equation (3) can be also approximated as Equation (4), for ψ_w_ in kPa:

(4) $$\Psi_{w}\approx 462T(\frac{e}{e_{0}}-1)$$

In this work, prior to each run, a water content equal to 0.30 L/L of soil was added to the soil, roughly corresponding to the soil water content after free drainage [4]. The soil water content was also expressed as relative to saturation, *i.e.*, the amount of pores filled with water relative to total volume of pores (it is also called degree of saturation).

**Figure 4.**
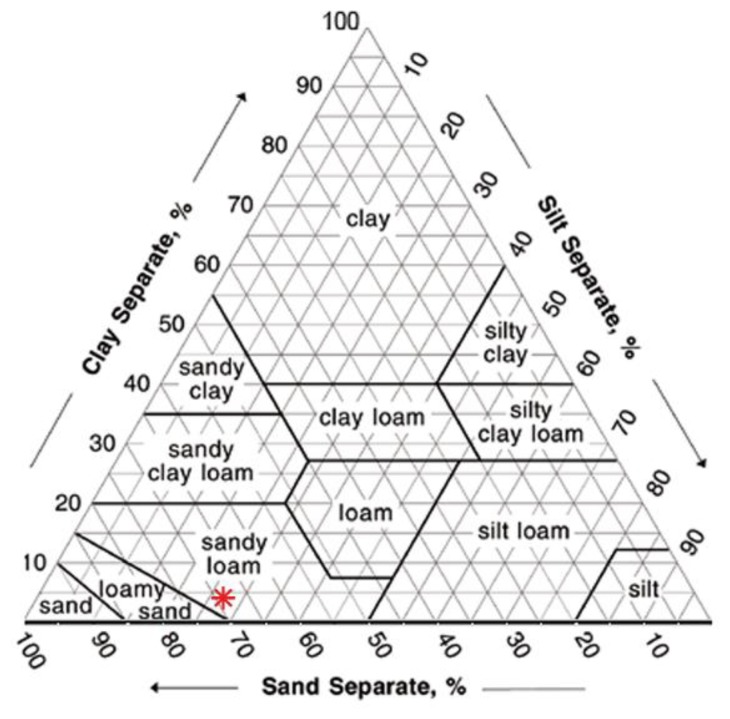
Soil texture triangle, showing the 12 major textural classes [39].

The soil used had a sandy loam texture (Figure 4). It was characterized by a high permeability and a good ability of water retention. Its chemical and physical characteristics are reported in Table 2. The soil used for the experiment was air-dried, sieved at 2 mm and homogenized to ensure repeatability of results.

The field test was performed on the same type of soil used for laboratory test, in the experimental site (called Tetto Frati) of the Department Agricultural, Forest and Food Sciences, in Carmagnola (near Torino, Italy).

A rectangular field (150 m in length and 7 m in width, for a total area of 1,050 m^2^) with a slight slope (0.5%) along the longest side to facilitate irrigation, and seeded with corn (in rows spaced 75 cm), was used for field sensors testing. During field water content monitoring, one irrigation was carried out on August 7th, by surface irrigation. The water content in soil was measured in different ways: by gravimetric method (soil sampling, oven drying at 105 °C and weighing, then multiplying by an independently- measured soil bulk density), by the TDR technique, by matric tension monitoring through tensiometers placed at 20 and 40 cm depth in a row and between two adjacent crop rows, and finally, by the sensors based on doped-hematite. All these measurements were carried out before and after irrigation for about one month; temperature and relative humidity of air, as well as rainfall, were measured.

**Figure 5.**
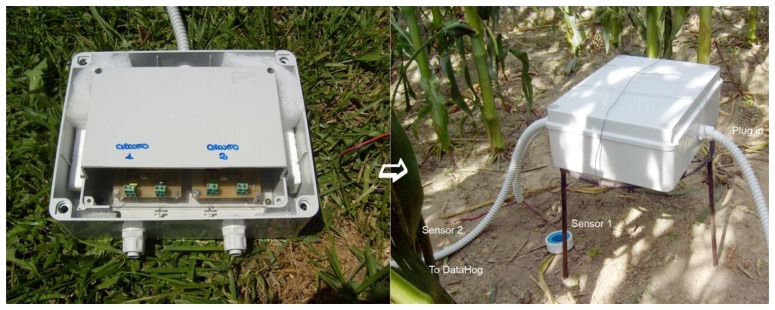
Field soil water content measuring system.

**Figure 6.**
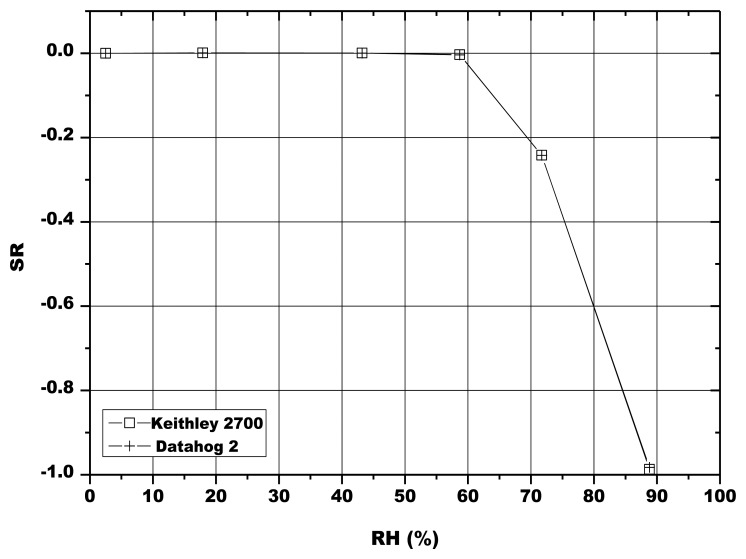
Comparison between the sensor response recorded by the digital multimeter. (Keithley 2700) and by the datalogger (Datahog 2) in function of the relative humidity.

For these tests, the electrical circuits that provided sensors alimentation were isolated from rainwater by a waterproof box (Figure 5). The sensors in the porous cups were inserted in soil at two different depths, at about 15 cm and 30 cm between two corn rows (Figure 5). Since it was not possible to use the digital multimeter *in-situ*, this instrument was substituted by a datalogger (DataHog 2, Skye Instruments). Before starting the field tests, a comparison between the multimeter and the datalogger was done, in order to verify a possible variation of the sensors electrical response. No significant difference in the sensor tension was then observed up to about 89 RH% (Figure 6).

## 3. Results and Discussion

### 3.1. Microstructural Characterization

The X-ray diffraction pattern (not shown here) of the Sr-doped hematite after the first thermal treatment at 900 °C for 1 h confirmed the presence of two phases: hematite (JCPDS card n.33-0664) and strontium iron oxide, SrFe_12_O_19_ (JCPDS card n.33-1340). Considering the initial 5 wt% equivalent SrO added to hematite, the powder should contain 90.24 wt% of hematite and 9.76 wt% of strontium orthoferrite.

**Figure 7.**
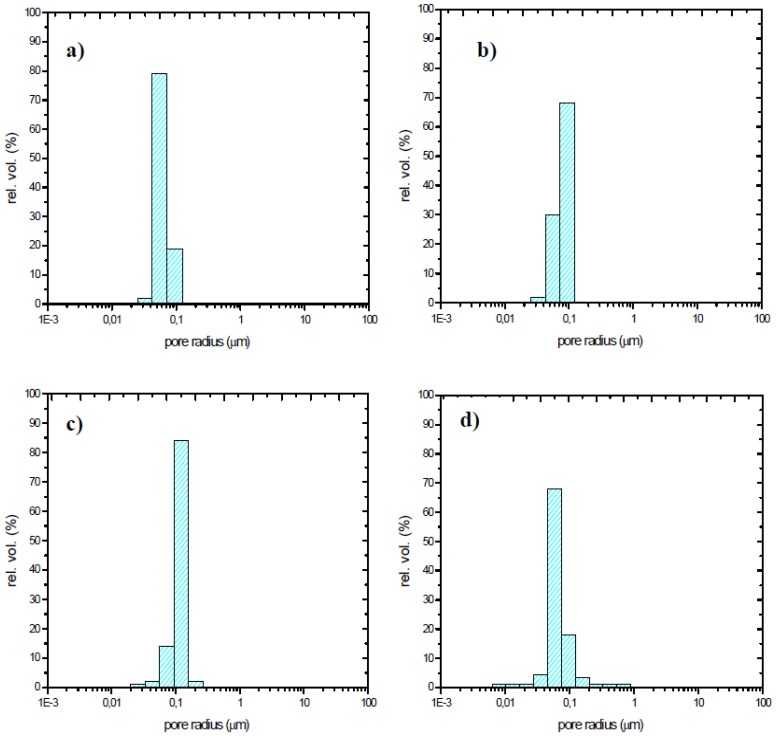
Pore size distribution of the samples with 5% SrO fired at (**a**) 800 °C, (**b**) 850 °C, (**c**) 900 °C and (**d**) 1,000 °C.

**Figure 8.**
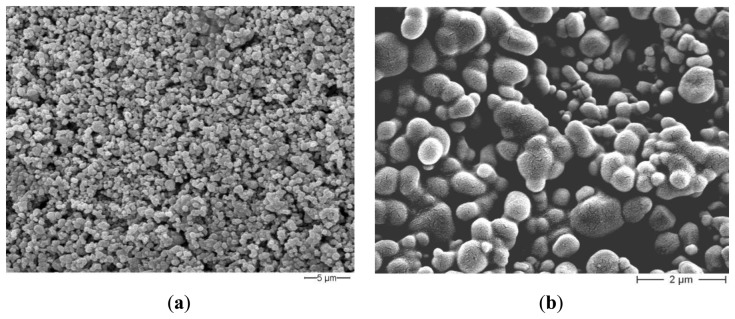
(**a**) SEM micrograph of Sr-doped hematite (×3,000); (**b**) FESEM micrograph of Sr-doped hematite (×15,000).

**Figure 9.**
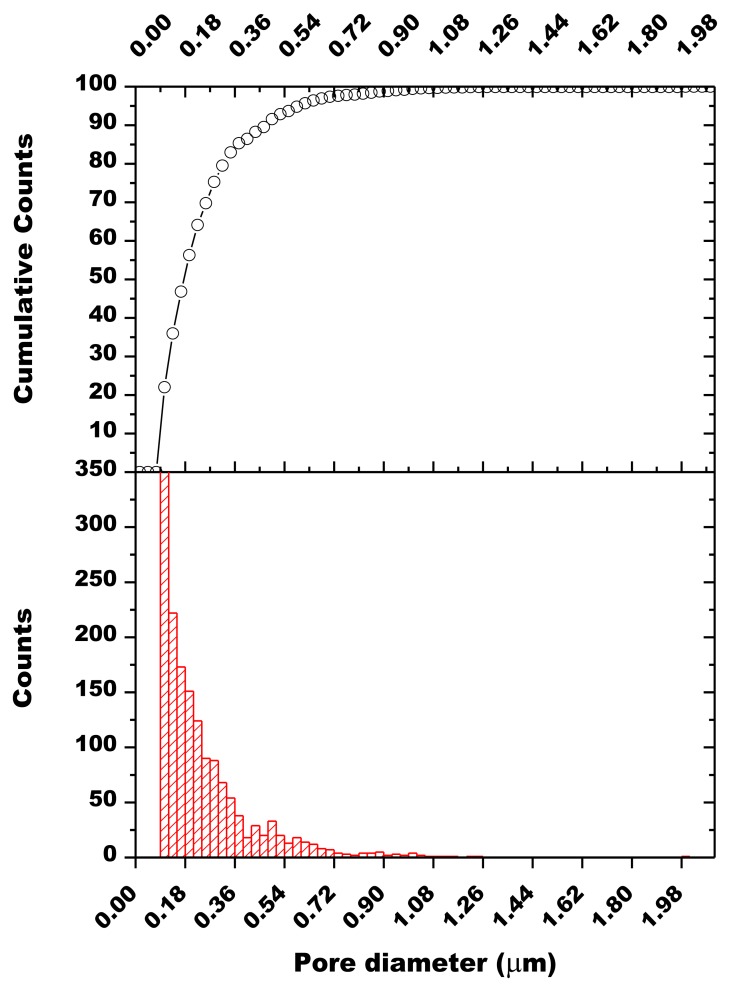
Pore size distribution in the screen printed film (1,593 objects measured).

The influence of the sintering temperature in the range 800–1,000 °C was studied by mercury intrusion porosimetry (Table 3): the opened porosity decreases together with the specific surface area when increasing the sintering temperature. The average pore radius is almost independent of the firing temperature in the range 800–900 °C and pore size distributions are similar, whatever the firing temperature, and are centered around 0.1 μm (Figure 7).SEM observations of the thick-films fired at 900 °C revealed a porous microstructure made of grains ranging from about 0.1 to 2.5 μm (Figure 8). Image analysis on seven SEM micrographs of a screen printed film allowed to determine an average porosity of 4.9 ± 1.7% and a mean pore diameter of 0.25 ± 0.18 μm (Figure 9).

### 3.2. RH Characterization in Air

**Figure 10.**
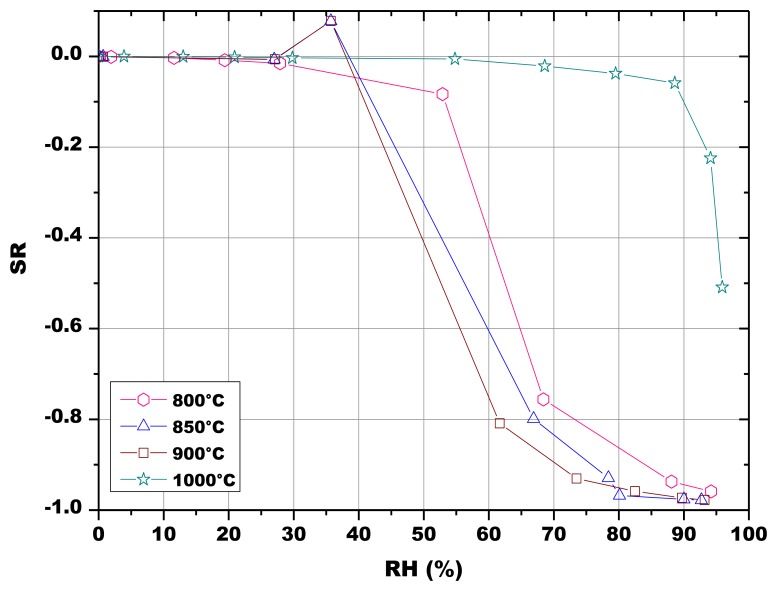
SR of the 5% SrO sensors (pellets) fired at different temperatures in function of RH at 23.6 °C ± 0.1 °C.

**Figure 11.**
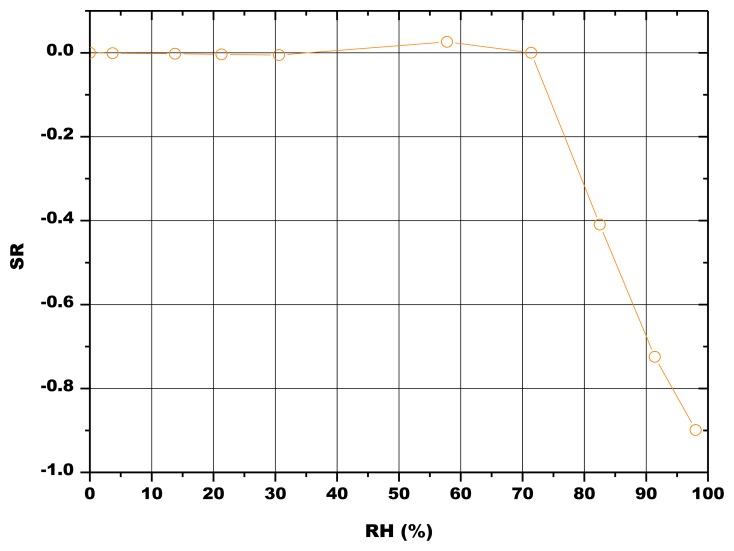
SR of the 5% SrO thick film sensor fired at 900 °C in function of RH at 22.3 °C ± 0.1 °C.

The initial resistances of the pellets were below 800 kΩ and rapidly decreased with increasing RH values, as illustrated in Figures 10 and 11. The sensors fired at 800 °C, 850 °C and 900 °C responded to water vapor over 30% RH, while the sensor fired at 1,000 °C responded only in the range 55–100 RH% (Figure 10). The response to humidity of the screen printed film was similar to the one of the pellet fired at 1,000 °C (Figure 11).

It is well known in the literature that water molecules chemisorb on the available sites of the oxide surface by a dissociative mechanism to form two hydroxyl ions for each water molecule [9,40]. These hydroxyl groups adsorb on the metal cations and the protons react with an adjacent surface O^2−^ group to form a second OH^−^ group. Once formed, this chemisorbed layer is no more affected by surrounding humidity. From spectroscopic experiments on defective single crystals surfaces [41], it is known that chemisorption in perovskites is likely to be completed at a water pressure of the order of 0.1 Pa. At temperatures lower than 100 °C, subsequent layers of water molecules are physically adsorbed on the first hydroxyl layer when relative humidity increases. Water molecules in the succeeding physisorbed layers are singly bonded, dissociate to H_3_O^+^ and form a liquid-like network. The conduction mechanism depends on the surface coverage of adsorbed water.

When only hydroxyl ions are present on the oxide surface, the charge carriers are protons, from hydroxyl dissociation, which hop between adjacent hydroxyl groups. When water is present, while surface coverage is incomplete, H_3_O^+^ diffusion on hydroxyl groups dominates, but proton transfer between adjacent water molecules in clusters also takes place. When the first physisorbed water layer is continuous, charge transport is governed by proton hopping between neighboring water molecules in the continuous film (Grotthus chain reaction). This mechanism means that higher resistivity of the oxides is observed at low RH values [9,40], as experimentally verified. When the pores are cylindrical with one end closed, condensation occurs in all pores with radii up to the Kelvin radius given by the Kelvin Equation (5), at given temperatures and water vapor pressures:

(5) $$r_{K}=\frac{2\gamma M}{\rho RT\ln(\frac{P_{s}}{P})}$$

where *r_K_* stands for the Kelvin radius, *γ*, *ρ*, and *M* for the surface tension, density and molecular weight of water respectively, and *P* and *P_s_* for water vapor pressures in the surrounding environment and at saturation, respectively [42]. Pores of minor dimensions are then first filled with condensed water respect to pores having more important radii: water vapor starts to condense at room temperature in mesopores of size 20 Å around 15% RH and continues to around 1,000 Å under a saturated atmosphere. At this time, the electrical conduction is likely to occur through the continuous water layers within the porous sample [43]. Moreover, at 20 °C and under atmospheric pressure, the mean free path of water vapor molecules decreases from 85.44 to 84.82 nm in dry air containing humidity traces and up to the water vapor saturation. The molecular entry into the pore is possible if the free path of the incident vapor molecule is less than the pore size [44]. The desired pore size should be comparable to the molecular free path of vapor at all humidity conditions, so that the molecules can impinge and adsorb on the pore wall [44]. Finally, in addition to the protonic conduction in the adsorbed layers, electrolytic conduction occurs in the liquid layer of water condensed within capillary pores, thereby resulting in an enhancement of conductivity. These mechanisms explain why SR, defined in Equation (1), tends to decrease with increasing RH, for all the prepared sensors (Figures 10 and 11).

The results reported in Figure 10 seem to indicate a greater availability of water adsorption sites at low RH values for the pellets heat treated at 800 °C, 850 °C and 900 °C. The loss of porosity and of specific surface area because of the higher firing temperatures at 1,000 °C (Table 4) could be responsible for the reduced sensitivity to humidity, as specific surface area is the principal microstructure feature for sensing humidity under low RH conditions, while mesopore volume dominates under high RH conditions [9,44]. This can be confirmed by the fact that the response to humidity of this last sample is close to the one of the thick-film fired at 900 °C (Figure 11). As a matter of fact, the screen-printed film evidenced a limited porosity (4.9% ± 1.7%, estimated by image analysis from SEM micrographs).

**Figure 12.**
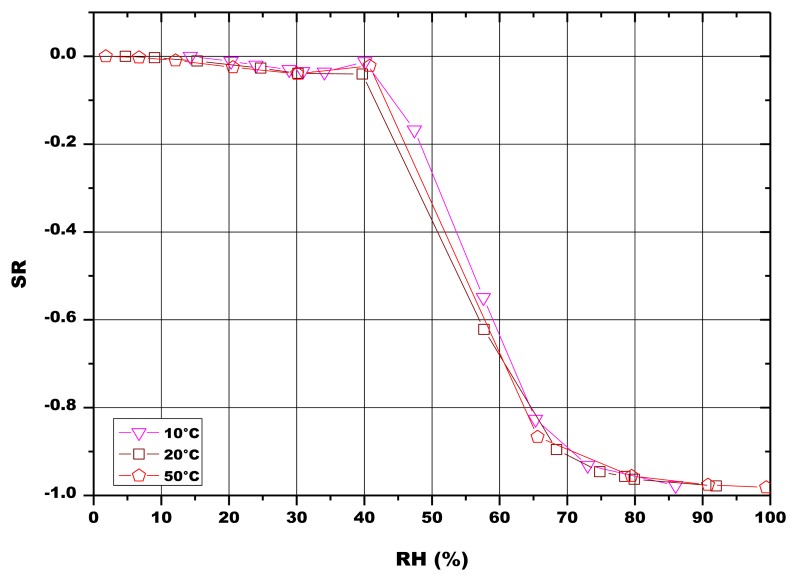
SR of the 5% SrO sensor (pellet) fired at 900 °C in function of RH, tested under different working temperatures.

Finally, the pellets fired at 900 °C were also tested at different temperatures, in order to study the influence of this parameter onto the sensors response (Figure 12). The results showed that the sensing material was almost insensitive to the working temperature, in the investigated temperature range (10–50 °C).

### 3.3. Soil Water Content Monitoring in the Laboratory

**Figure 13.**
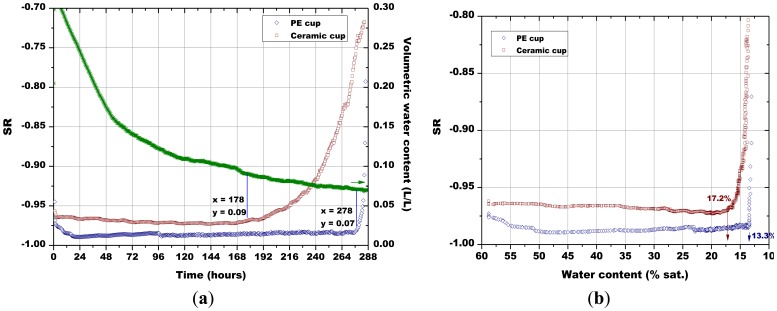
(**a**) Evolution of: the sensors response and of soil water content in function of time; (**b**) The sensors response in function of soil water content; influence of the PE and of the ceramic porous cup (soil temperature = 50.5 °C ± 0.8 °C, soil density about 1.30 g/cm^3^).

The first measurement (Figure 13) was devoted to the comparison between the monitoring system with the porous PE cup and with the porous ceramic cup above described, as the sensors cannot be inserted in soil without any protection. To this aim, two pellets of Sr-doped hematite fired at 900 °C were used. During the test, the sensors response reached quickly an absolute value close to 1, meaning that their resistance became very low, because they were in contact with an atmosphere saturated in water vapor (Figure 13a). After some days, their resistance started to increase progressively, indicating that the atmosphere in the porous cups contained less humidity. The sensors response were then plotted as a function of the soil water content (not shown here); these curves presented an almost constant region from the higher water content to near 0.09 L/L of water in soil. At that point, the sensor response decreased significantly. The intercept between these two regions was used to determine the minimum volumetric water content which led to a significant sensor response change. These volumetric water contents calculated for the different sensors were used to determine, on Figure 13a, the associated time after which the sensor's response changed significantly, as well as, the corresponding sensor's response. The ceramic cup exchanged humidity with the soil in a more progressive way with respect to the PE cup: the sensor response to relative humidity changes was observed after 178 h with the ceramic cup, against the 278 h necessary for the sensor in the PE cup (Figure 13a).

The water content in the simulated soil when the Sr-doped hematite went out of saturated water vapor atmosphere was 0.09 L/L for the sensor in the porous cup and 0.07 L/L for the one in the PE cup. Then, it was decided from now on to use only the ceramic protective envelope. Finally, no significant change in the sensor response has been observed with the porous ceramic cup mounted around the sensor, when the sensors were tested only under humid air. The plot of the sensors' response in function of the degree of saturation (Figure 13b) confirmed obviously that the ceramic porous cup favored the humidity exchanges with the soil, as the minimum water content which led to a significant change in the sensor response was higher with respect to the polymeric cup (17.2% and 13.3%, respectively).

**Figure 14.**
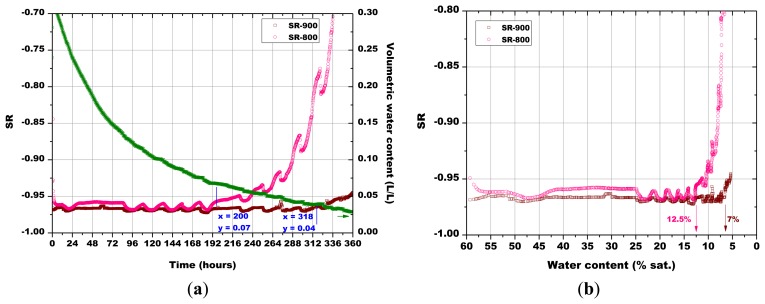
(**a**) Evolution of: the sensors response and of soil water content in function of time; (**b**) The sensors response in function of soil water content; comparison between the pellets fired at 800 °C and 900 °C (soil temperature = 41.5 °C ± 0.8 °C, soil density about 1.28 g/cm^3^).

**Figure 15.**
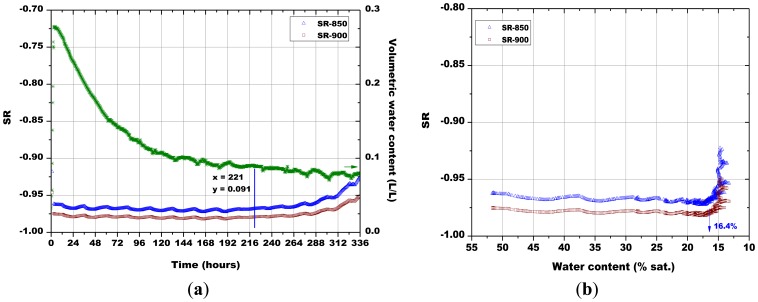
(**a**) Evolution of: the sensors response and of soil water content in function of time; (**b**) The sensors response in function of soil water content; comparison between the pellets fired at 850 °C and 900 °C (soil temperature = 38.7 °C ± 1.4 °C, soil density about 1.28 g/cm^3^).

**Figure 16.**
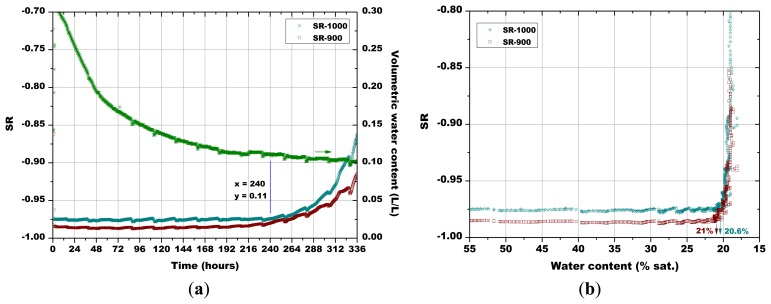
(**a**) Evolution of: the sensors response and of soil water content in function of time; (**b**) The sensors response in function of soil water content; comparison between the pellets fired at 900 °C and 1,000 °C (soil temperature = 45.0 °C ± 0.8 °C, soil density about 1.28 g/cm^3^).

**Figure 17.**
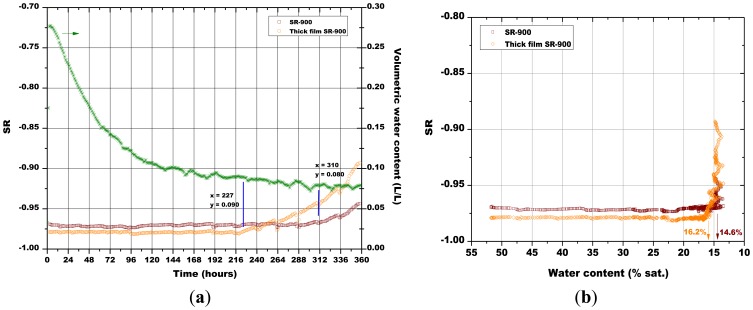
(**a**) Evolution of: the sensors response and of soil water content in function of time; (**b**) The sensors response in function of soil water content; comparison between a massive and a thick film sensor of Sr-doped hematite (soil temperature = 36.0 °C ± 1.0 °C, soil density about 1.28 g/cm^3^).

Subsequently, the influence of the sintering temperature (800 °C, 850 °C, 900 °C and 1,000 °C) on the Sr-doped hematite sensor response was investigated (Figures 14, 15, 16 and 17).

The last monitoring of the humidity content in the simulated soil was done with a pellet made of α-Fe_2_O_3_ + 5 wt% of SrO and a screen printed film based on the same material, both fired at 900 °C (Figure 17). The massive sensor gave a slower response than the thick film sensor to the variations of the water content in soil. In fact, the response of the pellet happened after 310 h and the one of the thick film sensor was recorded after 227 h, for soil water content of 0.090 and 0.080, respectively.

A certain dependence of the temperature variations was observed for all the sensors, as their response fluctuated in the same way as the temperature changed in the soil, as monitored by means of a Pt100 thermocouple: any decrease in temperature caused an increase of the sensor response and *vice versa* (Figures 14, 15, 16 and 17). These results can be explained by the fact that in a porous system as soil is, when temperature increases, the internal RH does the same, because the internal RH is linked to the meniscus curvature of water surface in pores which decreases due to a lowering in surface tension. Therefore, the sensor resistance goes down because of an increase in RH.

As explained above for Figure 13, the minimum water contents were determined for Figures 14 and 17, as well as the corresponding sensors response values. Finally, from the plot reported in Figure 10, the sensor's response was associated to the corresponding air relative humidity value. The best results were obtained with the pellet fired at 1.000 °C and with the thick film, for which RH values of 0.988 and of 0.996 were estimated, respectively. Finally, by means of Equation (3), the RH values were converted to a soil matric potential equal to −1,770 kPa and −570 kPa, respectively for the pellet fired at 1,000 °C and for the thick film, taking into account the average soil temperature measured during each run.

**Figure 18.**
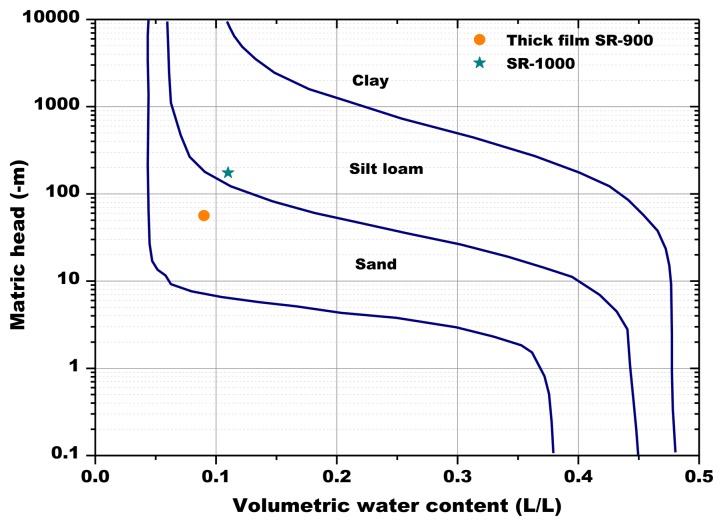
Example soil water retention relationships for three soil textures (adapted from [3]).

The volumetric soil water content determined by the Sr-doped-hematite based sensors is below the limit of the wilting point for a sandy soil (−1,500 kPa or −150 m, Figure 18), for the thick film.

The thick films response time were then determined, though, for the targeted application, this parameter is not crucial. To calculate this feature, the relative humidity was varied by steps, each of 30 min. The results (not shown here) evidenced that only negligible variations of sensor's response were observed after 1 min, indicating that the sensor's response time was quite fast in the investigated RH range. Recovery times were of the same order of magnitude: about 30 s were necessary for water molecules desorption when relative humidity changed from 92.4% to 10.3%. These results may be due to the large micropores, above 0.5 μm (Figure 8), that are necessary for rapid response to humidity changes [21]. The measured response and recovery times are shorter than the values reported in [21], for pure and Sn^4+^ or Mo^6+^-doped MgFe_2_O_4_, and in [28], for Li-substituted Mg-ferrite, and are comparable to the ones mentioned in [34] for Mn-Zn ferrites.

### 3.4. Preliminary Field Testing

**Figure 19.**
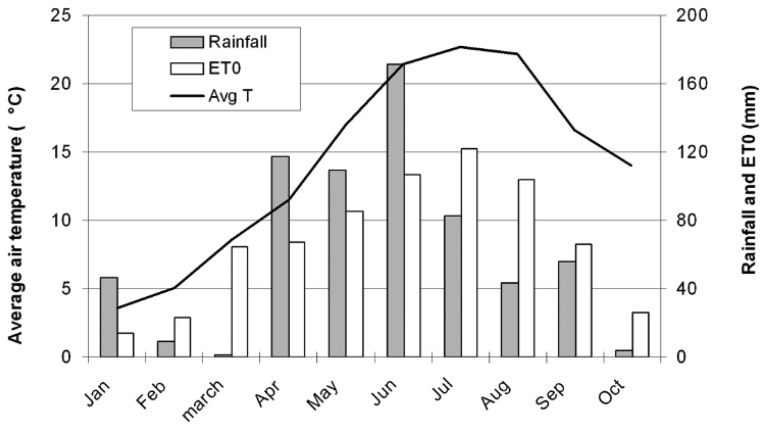
Average temperature, precipitations and evapotranspiration loss during the period January–October.

When the sensors were tested in field, precipitations were concentrated in April, May and June (Figure 19), with a peak in June with 171.4 mm. The reference evapotranspiration loss (ETO), is a term used to describe the sum of evaporation and plant transpiration from the Earth's land surface to atmosphere, it exceeded the natural water contribution in July and August requiring the irrigation of the field. In addition to the ETO, the precipitations were considered for analyzing all the data because the sensors and the electronic tensiometers detected all water contributions. The water content was measured before and after irrigation at two different depths and with two different methods: the gravimetric method and the TDR; the results were comparable and correlated (Table 4). A certain level of dispersion is expected, as although raw TDR measurements are often used in research, a soil - specific calibration is in fact required for precise measurements.

**Figure 20.**
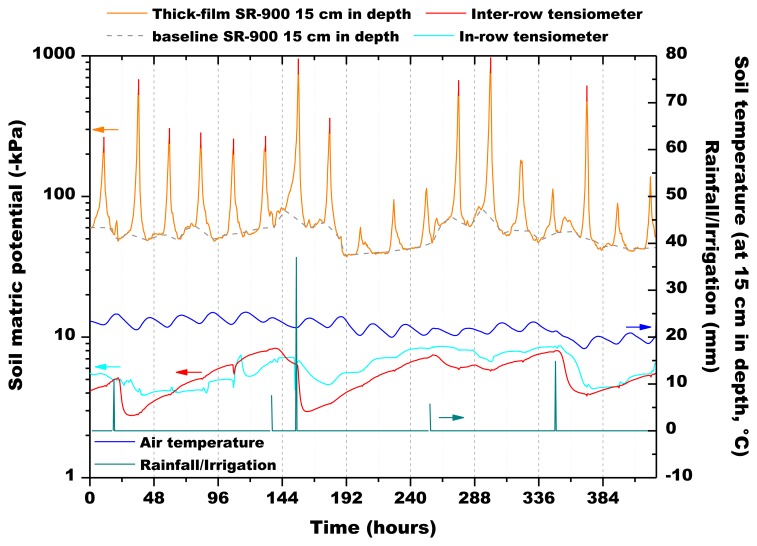
Field sensors response (origin of the curves is on August, 1st at 00:00).

During preliminary field testing, the matric tension varied in the range 280–900 kPa, which were values adapted for corn growth. The tensiometer at 40 cm in depth did not show any significant variation during monitoring. Nonetheless, it is important to mention here that the Sr doped-hematite sensors worked perfectly during the one-month monitoring. The sensor at 30 cm in depth, as the tensiometer, did not show a significant variation of the electrical response. On the contrary, the sensor at 15 cm in depth showed variations correlated to the water content variation (Figure 20). For the sake of comparison with the results of the two tensiometers, in Figure 20, the sensor's response has been converted to a matric potential by means of Equation (3), considering soil temperature at 15 cm in depth (average temperature during monitoring: 22.1 °C ± 1.7 °C).

In Figure 20, the dashed and the dotted vertical lines correspond to midnight (considering that the origin of the curves is on August, 1st at 00:00) each day and indicate minima in the sensor's response curve. On the contrary, the observed daily peaks are located in correspondence to the central hours of the day. These observations let us suppose that the humidity content in the porous cup was lower during the night respect to what happened during the day.

However, this effect could not be associated to soil temperature changes because during field testing the standard deviation of temperature (±1.7 °C) was similar to the one measured during laboratory runs (Figures 13, 14, 15, 16 and 17), and variations of sensors response due to temperature changes were only slightly evidenced. Therefore, these measurements could have been influenced by corn bioactivity because of water flows due to water consumption by plant roots (the sensors were inserted between two rows of plants). CO_2_ content in the soil [45] could also have influenced the measurements, as it is known in the literature that this gas can interfere with humidity monitoring [11].

If we consider the baseline of the peaks linked to the screen printed sensor response at 15 cm in depth, all the devices showed the same behavior to the water consumption by the plants (drying process) and by the natural water contributions (irrigation and precipitations). In the wetting process, soil water infiltrates in the liquid phase, while in the drying step, the vapor phase is dominant. In a first step, soil water evaporates rapidly and thoroughly near the soil surface due to the daytime temperature rise, the day after the rainfall event. In a second step, the soil water evaporates predominantly from the shallower depth. At this time, water vapor is supplied from the deeper soil. In a third step, by repeating diurnal variations of humidity, surface soil becomes dry due to the decreasing of the supply of water vapor from deeper soil [46]. All the instruments recorded the humidity changes in the soil. However, sensor response times were variable: the tensiometers tended to detect the wetting front before the screen printed sensors. Water moves through the soil profile at variable rates depending on the initial soil moisture status. The initial (pre-rainfall/pre-irrigation) soil moisture content was comparable for each of the sensors as all have identical irrigation (and rainfall) histories. They also have been installed in a quite identical relative position in the irrigation wetting zone. Given these factors it could be assumed that the sensors were in identical soil environments, however, soil is not a homogenous medium. Soil properties always vary even in the most uniform soil types [3,4]. Then, variation in soil properties (and the presence of preferential pathways) could contribute to the observed variation in response time.

Finally, installation may be another factor responsible for the observed differences in response time. The soil profile has to be disturbed to install a soil moisture sensor, particularly bulk density, which of course affects infiltration rates and consequently response times.

To conclude, both thick films, once removed from field testing, were again tested on the laboratory apparatus, and their response (not reported here) were very close to the one plotted on Figure 11, indicating that prolonged (one month) contact with humidity, and also probably with liquid water, did not alter their responses.

## 4. Conclusions

Five wt% strontium oxide-doped hematite is a suitable material to produce low-cost humidity sensors for soil water content determination: the material was sensitive in the range 75–100 RH% in air, and results in simulated soil demonstrated that the sensors' response were below the wilting point in a sandy loam soil. During field tests, the electrical signal of the sensors was correlated to the measurements performed with two tensiometers. It was then demonstrated that the sensor response was influenced by water content in soil, and probably also by plants' bioactivity.

The results also indicate the importance to evaluate and compare soil moisture sensors under different soil characteristics (texture, temperature, bulk density, and salinity) and under different moisture regimes, to check the influence of these features on sensor responses. The possible interference of CO_2_ during humidity measurements must be studied too. This would allow a better quantification of the accuracy of in situ measurements. In the future, different compositions could be investigated too, in order to increase the sensors response in the range 98–100 RH%.

## Figures and Tables

**Table 1.** Porous SDEC 2150 ceramic cup nominal features [38].

**Air Entry Value**	**1.5 10^5^ Pa**
Pore size	≈2 μm
Hydraulic conductivity	5.5 × 10^−7^ cm · s^−1^
Weight	30 g

**Table 2.** Chemical and physical characteristics of the soil used in this work.

**Tetto Frati**	
Coarse sand	7.5%
Fine sand	61.2%
Coarse silt	8.6%
Fine silt	18.2%
Clay	4.5%
pH (H_2_O)	7.6
CaCO_3 tot_.	28.8%
Organic content	1.18%
Bulk density	1.42 g/cm^3^

**Table 3.** Total opened porosity, average pore radius and specific surface area determined by MIP (average of two measurements).

**Sample**	**Total Opened Porosity (%)**	**Average Pore Radius (μm)**	**Specific Surface Area (m^2^/g)**
Hematite + 5 wt% SrO–800 °C (SR-800)	42.8	0.063	4.8
Hematite + 5 wt% SrO–850 °C (SR-850)	42.2	0.063	4.5
Hematite + 5 wt% SrO–900 °C (SR-900)	40.8	0.063	4.4
Hematite + 5 wt %SrO–1,000 °C (SR-1,000)	34.4	0.079	3.1

**Table 4.** Soil water content.

**Depth (cm)**	**Irrigation Event: August 7th**	

	**Water Content (% sat) Before**	**Water Content (% sat) After**
Gravimetric Method		

0–20	0.408	0.466
20–40	0.543	0.568

TDR		

20	0.283	0.328
40	0.390	0.445
